# Correction: Functionality integration in stereolithography 3D printed microfluidics using a “print-pause-print” strategy

**DOI:** 10.1039/d5lc90036k

**Published:** 2025-04-04

**Authors:** Matthieu Sagot, Timothée Derkenne, Perrine Giunchi, Yohan Davit, Jean-Philippe Nougayrède, Corentin Tregouet, Vincent Raimbault, Laurent Malaquin, Bastien Venzac

**Affiliations:** a LAAS-CNRS, CNRS 7 Avenue du Colonel Roche 31400 Toulouse France bastien.venzac@laas.fr; b Smartcatch 1 Place Pierre Potier 31100 Toulouse France; c MIE, CBI, ESPCI Paris, Université PSL, CNRS 10 Rue Vauquelin 75005 Paris France; d Institut de Mécanique des Fluides de Toulouse (IMFT, UMR 5502), Université de Toulouse, CNRS, INPT, UPS 2 Allée du Professeur Camille Soula 31400 Toulouse France; e Institut de Recherche en Santé Digestive (IRSD, U1220), Université de Toulouse, INRAE, ENVT, UPS 105 Avenue de Casselardit 31300 Toulouse France

## Abstract

Correction for ‘Functionality integration in stereolithography 3D printed microfluidics using a “print-pause-print” strategy’ by Matthieu Sagot *et al.*, *Lab Chip*, 2024, **24**, 3508–3520, https://doi.org/10.1039/D4LC00147H.

The authors sincerely apologise for providing the incorrect name of a reagent used in the protocol. In the main text, ‘Material and methods’, ‘Coverslip preparation’, the authors state “They were then immersed in a solution of ethanol (Micropur), 0.01% v/v of glacial acetic acid (Sigma-Aldrich) and 2% of (3-aminopropyl)trimethoxysilane (MAPTMS, Sigma-Aldrich) during 1 h”.

However, this should read as “They were then immersed in a solution of ethanol (Micropur), 0.01% v/v of glacial acetic acid (Sigma-Aldrich) and 2% 3-methacryloxypropyltrimethoxysilane (MAPTMS, Sigma-Aldrich), for 1 h”.

This error occurred due to both reagents being commonly abbreviated to MAPTMS.

The Royal Society of Chemistry apologises for these errors and any consequent inconvenience to authors and readers.

